# Exploring the relationship between video game expertise and fluid intelligence

**DOI:** 10.1371/journal.pone.0186621

**Published:** 2017-11-15

**Authors:** Athanasios V. Kokkinakis, Peter I. Cowling, Anders Drachen, Alex R. Wade

**Affiliations:** 1 Department of Psychology, University of York, York, United Kingdom; 2 Department of Computer Science, University of York, York, United Kingdom; University of Exeter, UNITED KINGDOM

## Abstract

Hundreds of millions of people play intellectually-demanding video games every day. What does individual performance on these games tell us about cognition? Here, we describe two studies that examine the potential link between intelligence and performance in one of the most popular video games genres in the world (Multiplayer Online Battle Arenas: MOBAs). In the first study, we show that performance in the popular MOBA League of Legends’ correlates with fluid intelligence as measured under controlled laboratory conditions. In the second study, we also show that the age profile of performance in the two most widely-played MOBAs (League of Legends and DOTA II) matches that of raw fluid intelligence. We discuss and extend previous videogame literature on intelligence and videogames and suggest that commercial video games can be useful as 'proxy' tests of cognitive performance at a global population level.

## Introduction

Games of strategy, such as chess or mancala, can be found across cultures and skilled performance in these games has been associated with intelligence [1–4] historically. Spitz formalized this connection with specific subpopulations, pointing out that performance in a wide variety of strategy games such as *Tic-Tac-Toe* or the *Towers of Hanoi* can be linked to mental ability [3,5]. He went on to suggest that strategy games tap a number of facets of intelligence: visualization of possible moves, short-term memory rehearsal and the ability delay immediate gratification to increase future rewards (for example, sacrificing a piece in chess in order to win the game in a later turn) [1]. Later studies consolidated the link between intelligence and game performance. For example, expert chess players have above average intelligence and that the correlation between skill level as approximated by rank and IQ scores (fluid and crystallised intelligence measurements) explains up to 30% of the variance [6–9].

This notion was extended to the domain of video games by Rabbitt et al. [10] who correlated scores from the Alice-Heim (AH-4) IQ test with performance in ‘*Space Fortress*’; an arcade-like single player game developed by psychologists [11–13]. While individual player IQs did not predict initial performance in Space Fortress, they *did* predict learning rates and, therefore, performance once players had engaged with the game long enough to become practised. More recent studies have suggested that IQ can be measured in a subset of simple single-player video games [14,15] as well as through tasks embedded in game-like environments [16]. In our current paper we extend their findings by asking whether we can establish a link between intelligence and performance in widely-played, commercial, team-based videogames with global reach.

More specifically, we focus on performance in a category of videogame that is played by millions of people: ‘Multiplayer Online Battle Arenas’ (MOBAs). MOBAs are action strategy games that typically involve two opposing teams of five individuals. Each individual controls one unit in a bounded map and the objective is to destroy the opponents’ base [17]. In comparison to the relatively specialized games analysed in some previous studies, MOBAs are, by some measures, the most popular games on the planet with an aggregate of at least 100 million registered active players. Findings based on these games are therefore important because they have relevance to the lives of a significant fraction of the global population. Their complexity also makes them intriguing targets for scientific investigation. While many previous studies have examined the cognitive effects of playing ‘First Person Shooters’ (FPS) [18–25], MOBAs have a reduced emphasis on hand-eye coordination but a far stronger dependence on memory, tactics and strategy which may, in turn, tap cognitive resources more closely linked to fluid intelligence.

Here we perform two separate studies performed using two independent video game datasets both of which address the relationship between intelligence and MOBA video game performance.

### Study 1

In **Study 1** we attempt to establish whether there is relationship between fluid intelligence and video game performance in MOBAs. We describe an experiment in which we measure psychometric factors related to intelligence in individual players under laboratory conditions and correlate these factors with players’ ranks in the popular commercial MOBA ‘*League of Legends’* (LoL) [26]. Specifically, we ask whether a common measure of Fluid Intelligence (scores on the WASI II Matrix test) correlates with LoL rank. Because working memory and fluid intelligence are highly related [27,28], we also tested players on a battery of WM tasks to ask whether WM itself was the key driver in performance.

One confound in our IQ/rank results could be players’ ability to work socially with other members of their team or to impute the motives of the opposing team members. Poor theory of mind (TOM) processing could therefore affect performance directly. In addition, TOM scores have also been shown to correlate positively with IQ and team performance in LoL after practice [29,30] and so any correlation between performance and IQ could potentially be explained by TOM. To control for this, we also presented our subjects with a test that measures aspects of TOM: The ‘Reading the Mind in the Eyes Test’ (MITE) [29,31]. We performed correlation and partial correlation analyses to determine whether scores on this test can explain the relationships we find between WASI II scores and performance.

### Study 2

The results from Study 1 suggest that there may be a correlation between IQ and video game rank. In order to address this question from another angle we used large data relating to video game performance to see if potential effects of Raw IQ can be detected. In particular, we ask whether performance in MOBAs follows the age profile that would be predicted if it correlated with raw Fluid Intelligence. We analyse data from two MOBAs (‘*League of Legends’* and *Defense of the Ancients 2’* (Dota 2) [32] which have more than 100 million registered unique players between them. We specifically asked whether performance in our these two MOBA datasets followed a trajectory that peaks in the early to mid-20s.

To control for age-related factors that may not depend on IQ, we compared these games with another popular genre: ‘First Person Shooters’ (FPS) in which players control a character from the ‘first person’ perspective and engage in combat within a simulated 3D world. Our comparison data come from two popular exemplars of the FPS genre: *Destiny* [33] and *Battlefield 3* [34] which have peaked at an aggregate of over 25 million registered players. We specifically use FPS games as a comparison because they appear to prioritise speed and targeting accuracy over memory and multifactorial decision making [35–37] and may therefore reflect a different set of cognitive performance characteristics (in particular, reaction times) that peak at earlier ages. However, all game categories considered here are ‘progressively complex’ [15,38]—vast array of possible responses are available at each time point and players are matched against opponents with approximately equal skill. In principle therefore, each of these games might potentially tap some aspect of fluid intelligence.

## Materials and methods

### Study 1: Fluid Intelligence and associated measures

#### Ethics

All participants in our laboratory experiments provided informed consent and approval for the study was provided by the ethics board of the Psychology Department of the University of York. All data were anonymized and participants were informed that they could withdraw from the study at any time.

#### Participants

Participants (N = 56, 51 males, mean age 20.5 years) were recruited via adverts from multiple sites within the UK in and around the Universities of Leeds, Essex and York. All subjects were experienced LoL players who had played a large number (>100) of both ‘ranked’ and ‘unranked’ matches. For more specific criteria and the advertisement please see S5 File.

#### Instruments

In this analysis we obtained psychometric test scores from subjects under laboratory conditions. We then compared those score with performance as measured by the subjects’ League of Legends rankings.

We used the WASI-II [39] Matrix Subtest (which is similar to Raven’s Matrices) as a standardized measure of fluid intelligence, along with three complex span working memory tasks (Symmetry, Rotation and the Operation Span task) that have been validated extensively; see S6 File [40–45].

Working Memory is closely related to fluid intelligence which we included as a complementary way of measuring cognitive ability [27,28,46,47]. We performed correlation analysis with a set of working memory measures to assess its relationship to the video game rank data. We also asked if a single underlying latent variable constructed from scores on the WASI and the three working memory tests provided a more parsimonious explanation for our results. Using IBM SPSS Amos (version 24, IBM Corp, NY) we ran a confirmatory factor analysis (CFA) and confirmed that a valid single factor could be constructed (see S3 File). However the correlation with our Rank scores was weaker (although still significant) and for simplicity we choose to present correlations with individual test scores here.

We also included the Mind in the Eyes Test (MITE) [31] which is designed to probe subject’s understanding of other people’s emotional states and which has been shown to correlate with intelligence [29]. In the MITE participants had to identify the emotion a face conveyed just from their eyes without hints from the rest of the face. Test administration was counterbalanced to eliminate order/presentation effects [48].

#### Rank

Online videogames such as the ones examined here provide detailed telemetry to the coordinating game servers. Companies such as Riot therefore have databases of real-time information about the behaviour and performance of game players.

We asked participants to provide their online nicknames so that we could access their game history and rank through a publicly-accessible website that interfaces directly to the Riot Games API database (https://euw.op.gg/).

Each player’s video game rank (computed from their position in an ELO-like ranking system similar to that used by the United States Chess Federation) was extracted from an online database [49]. In LoL players are divided into ranked ‘tiers’ with each tier having five ‘divisions’. A player’s position in these divisions and ranks depends solely on the ratio of matches won and lost over time [50] and not the performance within each match. Our participants’ rank ranged from ‘Silver Division 5’ up to the ‘Masters Division’ (see S1 File).

### Study 2—Age and performance

#### Ethics

We used existing data sources to ask whether performance in different types of games followed the age-dependent trajectory that would be expected if it was highly correlated with fluid intelligence. No player-identifying information was present in any of the datasets and data acquisition procedures were approved be a separate application to the University of York Psychology Ethics Committee. More details on player demographics are presented in Table 1.

**Table 1:** Player numbers and ages (in years) for the four games in our analyses.

	N	Minimum	Maximum	Mean	Std. Deviation
BF3	8743	13	40	23.67	6.53
Destiny	1669	13	40	24.18	6.32
Dota 2	286	13	40	23.11	4.02
LoL	17861	13	40	20.49	5.07

#### Data sources

All four videogames use the ratio of historical wins to losses as a primary metric for the ‘Matchmaking Ranking’ (MMR) score which we analyse here. MMR is a dynamically-updated measure of player performance that depends solely on the win/loss history of each player and the rank of their opponents. It should be noted that Divisions and Tiers in League correspond to an MMR range that is hidden from the user but provided to us by Riot Games.

***League of Legends***: A snapshot of LoL player ranks was provided by Riot Games (Riot Games, a subsidiary of TenCent Holdings, Los Angeles, CA). Other aspects of this dataset have been analysed in a previous paper [51].

***DOTA II***: A dataset from casual players who spectated at the ‘International 5 Dota 2 Tournament 2015’ was provided by the education analysts at Foundry 10 [52–54] and Valve (Valve LLC, Bellevue, WA).

***Destiny***: The anonymized Destiny dataset were obtained from the developer, Bungie (Bungie,Inc. Bellevue, WA), with age data from a public online survey of approximately 1700 Destiny players who participated on a voluntary basis.

***Battlefield 3*:** Anonymized Battlefield 3 (Electronic Arts, Redwood City, CA) data were obtained from Tekofsky and colleagues [55,56] and is available through their website (http://www.psyopsresearch.com/download/). In our analysis we used the data from the structure stats.global.elo.

#### Data analysis

Fluid intelligence changes with age [57–59]. Here, we asked whether performance in four different games had shared similarities in their aging profiles. Our hypothesis was that because MOBA performance correlates with fluid intelligence, it would follow an age trajectory similar to that seen in population-level raw IQ scores—peaking in the early to mid-twenties with a decline thereafter [57,60–62]. We chose the first person shooters *Destiny and BF3* as controls since performance on these game might be expected to correlate more with reaction time and therefore peak earlier in the lifespan [63]. MMR performance measures are available for each of these games but the absolute scaling of this ratio variable differs between games. To enable a direct comparison between the four games (*Dota 2*, *LoL*, *Destiny* and *BF3*) we Z-scored the MMR distributions for each game separately by removing the means and scaling by the standard deviations. We then separated these normalized within-game scores into three age groups designed to span the point at which raw IQ scores begin to decrease (13–21, 22–27 and 28–40 years old). The lower end of this range was a product of the registration requirements of the games we used. The high end was imposed to ensure that age bins were standardised across games: one of our datasets (DOTA II) had very few participants over this age cutoff. We note that Tekofsky et al [55] adopt a similar strategy for almost identical reasons.

Datasets for both studies are available on the Open Science Framework website https://osf.io/dsbx4/.

## Results

### Study 1—Raw fluid Intelligence scores and player Rank

**Fig 1:**
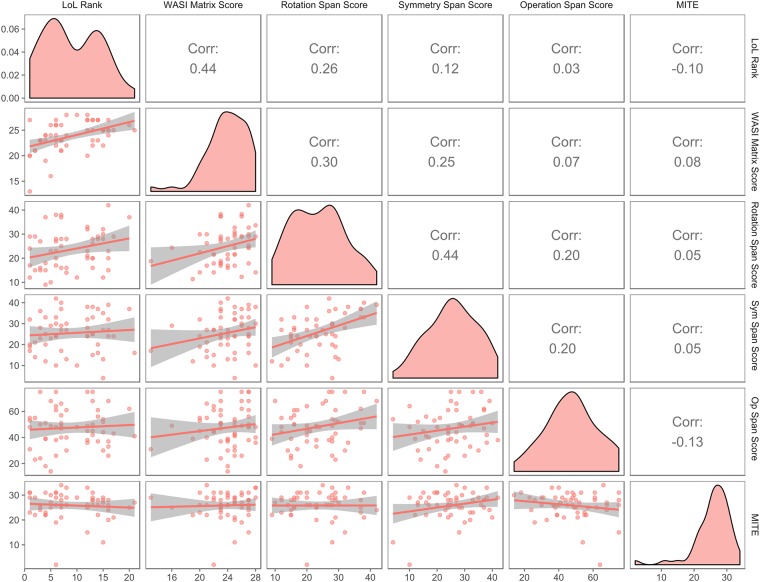
Cross correlations between variables of interest. The leading diagonal shows the distribution of the data. Numbers above the diagonal show the non-parametric cross correlation coefficient. Scattergrams of the data with best fit lines and error limits are shown below the leading diagonal. There is a moderately-sized and highly significant correlation between WASI-II Matrices and Rank (r_s_ = .44, p = .001) and a weak but significant correlation between Rank and Rotation Span score with *r*_*s*_ = .*26*, p < .05. The correlations between Rank and OSPAN and MITE task scores were not significantly correlated with with r_s_ = 0.3, p = .43 *and r*_*s*_ = -.*01*, *p* = .*242* respectively.

Because our data were not normally distributed, we computed the non-parametric ‘Spearman *rho’* correlation (Table 2) between Rank and performance on the standardized psychometric tasks (for more information about Ranking and alternative coding see S4 File). We found that fluid intelligence as measured by the WASI II Matrix Reasoning Subtest, correlated significantly with rank (nonparametric rank correlation: r_**s**_ = .44 (95% CI [.24 .60], p = .001)–See Fig 1. Importantly, we found no significant correlations between rank and scores in the MITE task and the partial correlation of WASI II scores with rank controlling for MITE was not significantly different to the initial correlation without accounting for MITE. Similarly, we found only a weak correlation between rank and a tests of visuospatial working memory.

**Table 2:** Non-parametric correlations between variables measured in Study 1.

			WASI II Raw Score	League of Legends Ranking	Rotation Span Score	Symmetry Span Score	Operation Span Score	Mind in the Eyes Test Score
Spearman's rho	WASI II Raw Score	Correlation Coefficient	1.000	.440**	.303*	.247*	.071	.082
		Sig. (1-tailed)	.	**.000**	**.014**	**.036**	.302	.275
		N	56	56	52	54	56	55
	League of Legends Ranking	Correlation Coefficient	.440**	1.000	.260*	.117	.025	-.096
		Sig. (1-tailed)	**.000**	.	**.031**	.199	.427	.242
		N	56	56	52	54	56	55
	Rotation Span Score	Correlation Coefficient	.303*	.260*	1.000	.440**	.198	.050
		Sig. (1-tailed)	**.014**	.031	.	.001	.080	.365
		N	52	52	52	50	52	51
	Symmetry Span Score	Correlation Coefficient	.247*	.117	.440**	1.000	.197	.050
		Sig. (1-tailed)	**.036**	.199	**.001**	.	.077	.361
		N	54	54	50	54	54	53
	Operation Span Score	Correlation Coefficient	.071	.025	.198	.197	1.000	-.131
		Sig. (1-tailed)	.302	.427	.080	.077	.	.171
		N	56	56	52	54	56	55
	Mind in the Eyes Test Score	Correlation Coefficient	.082	-.096	.050	.050	-.131	1.000
		Sig. (1-tailed)	.275	.242	.365	.361	.171	.
		N	55	55	51	53	55	55

The Rotation Span scores were weakly but significantly correlated with Rank (r_s_ = .26, *p <* .*05)*.

** Indicates correlations significant at p < .01 levels;

* Indicates correlations significant at p < .05 levels (1-tailed).

Outliers are always a concern in correlational analyses. To address this, we computed the Cook’s Distance for all points in the MMR vs WASI II analysis. The highest Cook’s distance was found for the subject with the lowest WASI II score. However, the Cooks’ Distance for this subject was .59 (well below the .70 threshold for our sample) and the correlation is virtually unchanged r_s_ = .435, p < .001 even if this player is excluded.

### Study 2—Performance as a function of age

**Fig 2:**
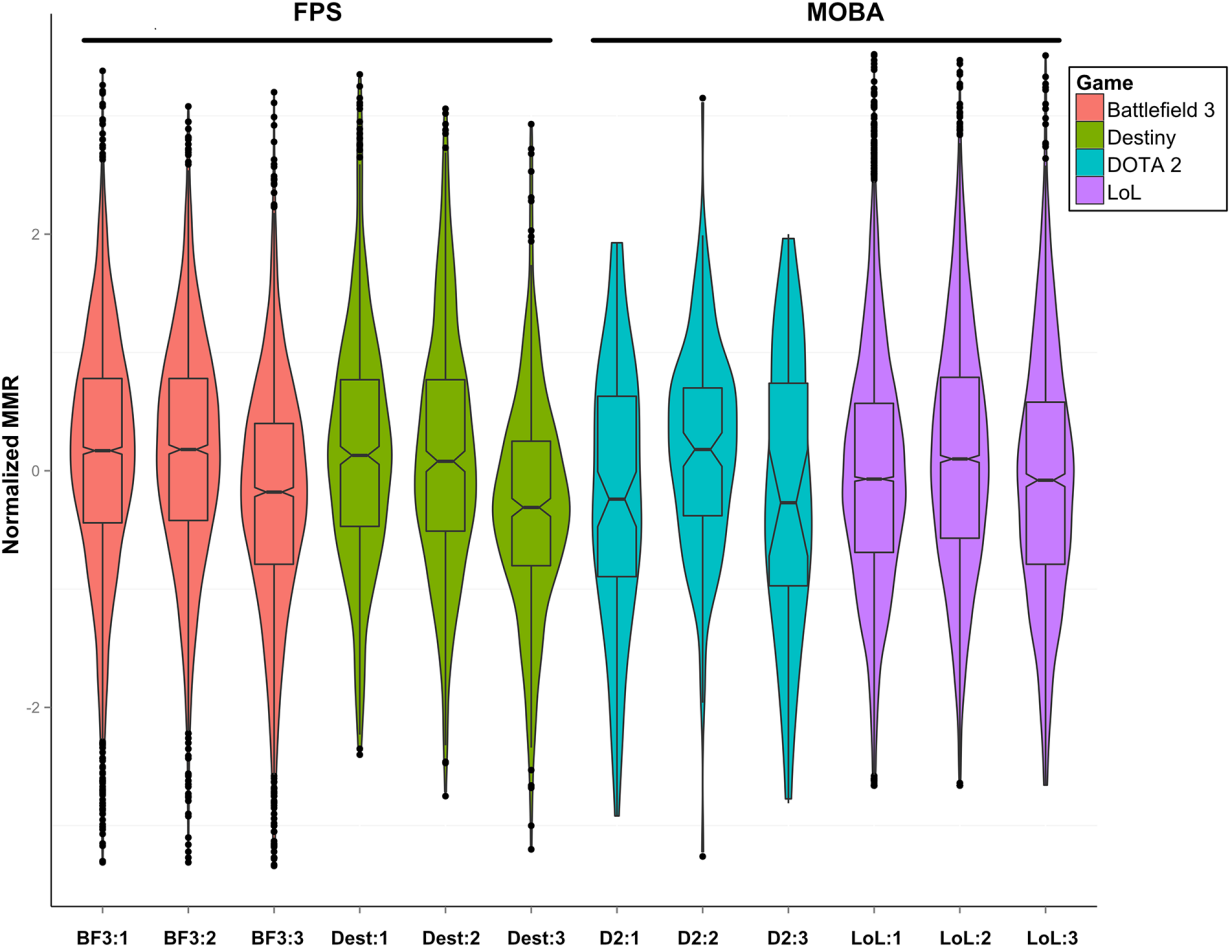
Age profiles of MMR in four different games. Three age groups for each game are plotted: (1) 13–21, (2) 22–27 and (3) 28 years an over. In two popular ‘First Person Shooter’ games (Battlefield 3 and Destiny), performance decreases monotonically with age following a ‘high, high, low’ profile. In comparison, two of the most popular multiplayer online battle arena (MOBA) games exhibit a ‘low, high, low’ profile suggesting that performance peaks in the mid-20s. Distributions whose boxplot notches do not overlap are different at p < .05.

Boxplots of the age-grouped MMR data are shown in Fig 2. Visual inspection shows that performance scores in the MOBA and FPS games follow a different age trajectory and ANOVA analysis of the data with planned comparisons confirms this observation. We are reporting Welch’s F due to the lack of homogeneity of variance.

There was a significant effect of age on MMR for Destiny (*F*(2,1076.55) = 40.21, *p* < .001), for Battlefield 3 (*F*(2,5185.93) = 122.87, *p* < .001), for Dota 2 (*F*(2,90.9) = 5.19, *p* < .05) and LoL (*F*(2,4114.37) = 57.44, *p* < .001. All games showed a significant difference between the middle group and the last group indicating that performance in general falls off after the mid 20s. Performance of the middle group when compared to the older group was significantly higher for Destiny, *t*(990.7) = 3.82, *p* < .001, d = 0.45, for BF3 *t*(4.609.5) = 8.63, *p* < .001, d = 0.4, for LoL, *t*(5760.8) = 9.59, *p* < .001, d = 0.17 and for Dota 2 *t*(113.22) = 2.93, *p* = .002, *d* = 0.38. Only the MOBAs ***also*** showed a significant increase (p < .001) between the first and second age group. This increase is consistent with the hypothesis that performance in MOBAs (but not FPS games) is correlated with fluid intelligence which also exhibits this age profile. For more detailed statistics and tables see S2 File.

Overall, we found that MOBA-genre performance profiles followed a ‘low,high,low’ pattern where performance peaked in the 22–27 year old age group. In comparison, FPS performance followed a ‘high,high,low’ pattern suggesting that younger players had a relative advantage in this genre and that performance decreases monotonically with age.

## Discussion

What can these results tell us about the link between commercial MOBA video games and Fluid Intelligence?

The literature around video games, psychology and neurophysiology (much of which focuses on FPS games) is extremely diverse (see Palaus 2017 for a review [64]). Green and Bavelier’s work in the early 2000s identified perceptual effects of FPS play and later studies extended this to attentional effects and cognitive tasks such as response inhibition, task switching and working memory [18–21]. While extended FPS play may lead to improvements in visuospatial processing [22] (although see [65–67]), the same subjects may also exhibit reduced ability to process emotional stimuli [25]. Finally, extended FPS play may reduce and/or increase cortical gray matter thickness and cortical connectivity [23,24,68,69] depending, in part, on the game strategies adopted.

These observations are important but the field is largely focused on the question of whether video game practise generates cognitive or perceptual benefits that transfer to other domains. This focus is due to several factors: Clinicians and health scientists are, understandably interested in the potential that video games may hold for neurorehabilitation, educators and parents are interested in the long-term effects that video game play may have on young people and neuroscientists see the extended training and perceptual measurements that video game play affords as an opportunity to learn more about relatively mature fields such as perceptual leaning.

Although our data indicate a link between intelligence and video game performance, the relationship is correlational and so the causality is unclear. One possibility is that rather than games modifying cognition, learning to play video games depends on the same cognitive resources underlying performance on intelligence tests. There is some support for this from previous research by, for example, Rabbitt *et al* (9) who showed that intelligence correlated with practised performance on “*Space Fortress*”: a simple, non-commercial video game designed by psychologists. This link is also supported by recent work showing that the rate of early learning in an online video game and the stable final performance are correlated, lending support to the idea that a single factor (presumably related to cognitive capacity) underlies both metrics [70].

Importantly, Stafford and Dewar’s work [70] suggest that cognitive capacity is assayed by a final, stable performance metric which is ultimately *invariant* to increasing practice. This correlation between performance after large amounts of practise and cognitive capacity is further supported by Adams and Mayer [71] who identified a correlation between scores in a first-person shooter (*Unreal Tournament*) and two mental rotation tasks in non-videogame players (Shepard-Metzler & paper-folding). Finally, similar results have been observed by Bonny & Castaneda [53] who that number processing ability not only correlates with Dota 2 MMR but is also predictive of MMR improvement over time.

In principle, the correlation might arise because playing video games causes an increase in intelligence. Although IQ scores are believed to be relatively stable [72], training on action video games does improve visuospatial performance [73] and the general mechanism (an improvement in probabilistic inference based on visual input [74] could, potentially, translate to a wide range of cognitive tasks. Addressing this possibility robustly would require a set of large-scale longitudinal experiments and is beyond the scope of the current study.

We note that we did not find a correlation between the processing aspects of the complex span task and MMR in Study 1. This might indicate that MMR depends most critically on the working memory aspects of these tasks. However, we also note that there was little variance in the processing scores—all subjects tended to score well in these tasks. It is possible therefore that a correlation between processing and MMR might emerge in a larger, more diverse sample population.

### Age

The final possibility is that some third factor is driving variance in both intelligence and video game expertise. One candidate is age. Raw (un-normalized) fluid intelligence scores usually peak in the mid-20s [60]. This also appears to be the approximate peak of video game performance in MOBAs that depend on a mixture of memory, tactics, strategy and reaction time (Fig 2) while games that emphasise more reaction times and hand-eye coordination (for example, FPS-type games) appear to advantage younger players. However, while we did find a significant correlation between WASI II scores and age in Study 1 (r = .28, *p* = .035) our LoL data showed no correlation between expertise and age and a partial correlation of expertise with WASI II accounting for age was still highly significant (*p* = .001, r = .45).

Age may also correlate with practise: older players may have had more time to practise any particular game (although it is also possible that older players are more restricted in the amount of free time that they can devote to game play). To examine whether pure practise effects determine rank, we examined the relationship between rank and games played in our large (N>17000) dataset of LoL players. After the initial learning stage during which the players attained a relatively stable rank, the magnitude of the correlation between games played and expertise (indicated by MMR) was r = .02. While still significant (p < .001) this suggests that games played explains only a small amount of the variance found between experienced players [53,75]. This agrees with Stafford and Dewer’s finding that final, stable performance levels are determined largely by the rate of learning in the initial phase rather than the total number of games played overall [70].

### Comparison with previous findings

The size of the effect we find in our correlation between rank and fluid intelligence (r_s_ = .44) is slightly smaller than those reported by other groups. For example, Foroughi et al [16] report a correlation coefficient of .65 between IQ and performance in a custom-made game based on Portal 2 while Quiroga et al [14] report a remarkable correlation level of .96 adjusted variance explained (equivalent to a correlation coefficient of around .98) in a study using games from the ‘Big Brain Academy’. These results confirm that it is possible to measure proxies of fluid intelligence using computer based tests and we have no doubt that some single player puzzle games like Portal test both abstract problem solving and visuospatial working memory. In both cases however, the games used were specifically selected (or designed) beforehand to probe IQ. In the case of the Quiroga battery in particular, the Big Brain Academy ‘brain training’ games resemble ‘gameified’ intelligence tests and the degree of of correlation with actual tests is, perhaps, less surprising (see also Banquied 2013 [76]). Here we describe a set of correlations that are remarkable because they are based on data from an unmodified, widely-played commercial video game that has no *a priori* links to intelligence testing. These data may also be noisier than the single-player data obtained by the groups discussed above because of the multiplayer nature of the MOBA genre—the outcome of each game depends on a team effort and the proficiency of the opposing team contributes additional variance.

We recognize that although fluid intelligence seems to be one factor in obtaining a higher MMR, it does not explain all the variance—practice, dedication and learning must still confer significant advantages [6,7]–particularly during the early stages of skill acquisition. This effect is mitigated to some extent in Study 1 by the fact that all our subjects were relatively well-practised (over 100 games *excluding* casual and unranked matches) and had demonstrated a willingness to engage with the game intellectually over a long period of time.

### Theory of mind

Finally, we found no correlation between performance in LoL and the MITE test which is probe of a subject’s ability to perform theory-of-mind tasks [31]. This was unexpected: MOBAs are social games and we believe that the ability to model the motives of other players enhances performance. In addition, scores on the MITE task have been shown to correlate with IQ [29] and Engel et al. [77] showed that MITE scores predict performance in cognitively demanding tasks such as solving Sudoku puzzles. We conclude that, at least in our relatively small sample population, any weak correlation between MITE and intelligence scores that may exist may be swamped by other factors. In addition, if the relationship between performance and MITE score is driven by subjects with poor TOM, we may not have sampled an MITE dataset with sufficient variance over the low end of the range to expose the effect.

### Sample size

The two studies presented here have very different sample sizes. For Study 1, N = 56. For Study 2, we have many thousands of data points. The two analyses therefore have different strengths and weaknesses. In Study 1, although N is small, the data are collected under controlled laboratory conditions using standardized instruments. Although the relatively small sample size cautions us that this work is still exploratory, the large effect size and strong significance are encouraging. The data from Study 2 *are* collected from larger cohorts but the provenance of each data point is less certain. Issues such as selection bias are potentially problematic and we expect to find some noise in measures such as age due to participants deliberately or carelessly reporting false information—although there is evidence that large web-based samples such as these can be relatively reliable [78]. The hope with large datasets such as these is that the huge number of participants more than compensates for the increase in noise caused by the sampling methods. An important challenge for future research is to test this assumption by broadening the use of validated tests to a wider subject group—perhaps through careful use of online crowdsourcing platforms [79].

## Conclusion

We propose that videogame expertise in commercial MOBAs correlates with fluid intelligence and the developmental trajectory of expertise mimics that of fluid intelligence across adolescence and early adulthood [58,60,80]. A decline in fluid intelligence and working memory has been linked with the expression of a number of diseases as well as with healthy aging [81–83]. The specific MOBA genre is remarkable in the sense that it already engages a vast number of players across the globe but more generally, complex, socially-interactive and intellectually demanding video games are now ubiquitous and generate a constant stream of performance data that can be normalized against millions of other players. If MOBAs in particular, or even video games in general offer a robust insight into cognitive function, they may be used to study cognitive epidemiology at a massive scale—instantly overcoming existing issues with small sample sizes [83] and potentially allowing us to examine dynamic changes in performance at a population level in almost real time.

## Supporting information

S1 File Data pre-processing. (PDF)

S2 File Analysis of variance. (PDF)

S3 File Confirmatory factor analysis. (PDF)

S4 File Correlations. (PDF)

S5 File Advert and recruitment. (PDF)

S6 File Tasks. (PDF)
