# Supplementary material for: Exploring the relationship between video game expertise and fluid intelligence
Source: PLoS One. 2017 Nov 15;12(11):e0186621. doi: 10.1371/journal.pone.0186621 (PMC5687598; doi:10.1371/journal.pone.0186621)
Supplement: S1 File — (PDF) [file pone.0186621.s001.pdf]

Supplementary Material – 1

Data pre-processing

ELO/MMR ratings

After 10 placement games LoL players are assigned a Tier and a Division based on their win/loss performance. They subsequently move up and down on that Division and move between Tiers based on their win ratio. Tiers and Divisions therefore correspond to MMR ranges. In addition, there may be non-linearities in MMR within and between Tiers and data in our relatively small sample do not pass standard tests for normality. For these reasons, we show parametric statistics here only for visualisation purposes and all correlations are computed using Spearman's rho. We note that the results from parametric and non-parametric analyses are almost identical.

Study 1

Rank Nonparametric correlations

We treat our data as parametric for visualisation purposes. However, League Tiers and Divisions are not normally distributed and thus we opted for non-parametric calculations throughout our analysis. We note that the results from parametric and non-parametric analyses are almost identical.

Study 2

All distributions were inspected for outliers and we then used Tukey's outlier technique (k=2.2) to identify other candidate points. Here we provide illustrations of the distributions before and after outlier rejection.

Note all these terms in the x-axis (ELO, Matchmaking Rating, Combat_PVP) are interchangeable (different naming conventions depending on the company) and they use win-ratio as a primary determinant of a player's "skill-level".

League of Legends

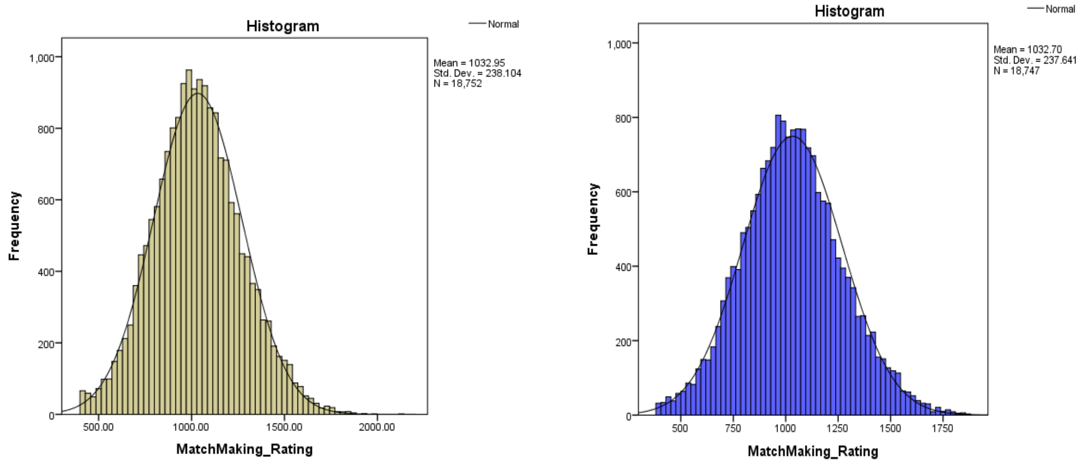

S1 Fig 1 Histograms of League of Legends MMRs before (Left) and after (right) outlier rejection

Battlefield 3

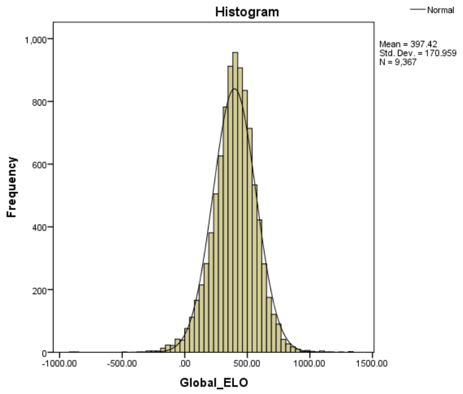

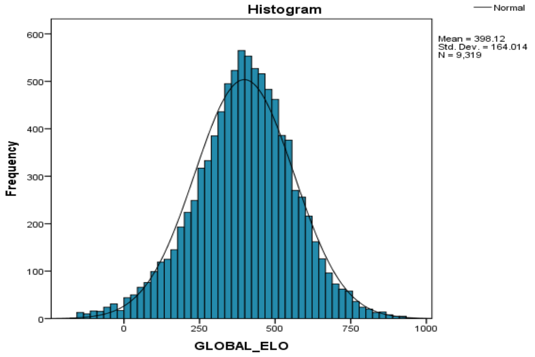

S1 Fig 2 Histograms of Battlefield3 ELOs before (Left) and after (right) outlier rejection

Destiny

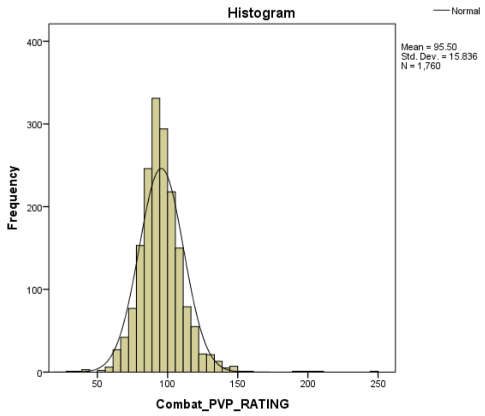

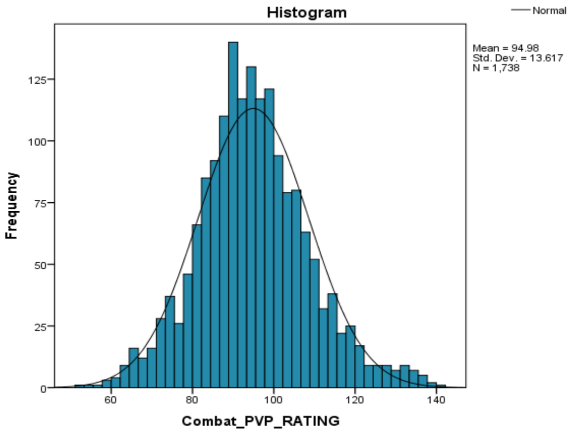

S1 Fig 3 Histograms of Destiny PVPs before (Left) and after (right) outlier rejection

DOTA 2

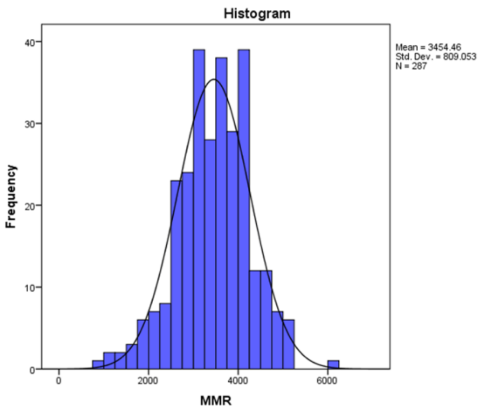

S1 Fig 4 Histograms of DOTA2 MMRs. No outliers were detected in this dataset
