# Supplementary material for: Exploring the relationship between video game expertise and fluid intelligence
Source: PLoS One. 2017 Nov 15;12(11):e0186621. doi: 10.1371/journal.pone.0186621 (PMC5687598; doi:10.1371/journal.pone.0186621)
Supplement: S2 File — (PDF) [file pone.0186621.s002.pdf]

Supplementary Material – 2

Analysis of variance

One way ANOVAs

Homogeneity of Variance

Only Battlefield 3 passed the test for homogeneity of variance (Levene's tests – see below). Although ANOVAs are robust to small violations of this assumption, we also report tests that do not assume equal variances. Results are qualitatively identical for all games.

Contrast Coefficients

We performed two planned contrasts as functions of age band on our ANOVA data to test for effects of age. The first contrast

[0 1 -1]

asks whether there is a significant difference between the last ('elderly') age band and the mean of the second group. Effectively, it asks whether we see a general age-related fall-off in all games after the mid-20. We hypothesized that all games would show this effect because both IQ and reaction-time measures begin to decrease after the mid-20s.

The second contrast

[-1 1 0]

asks whether we also see a performance advantage in the mid-20s compared to the teens. We hypothesized that we would see this effect for MOBAs (which, appear to depend strongly on fluid intelligence) but not for FPS games (which may depend more on rapid reaction times and hand-eye coordination).

GAME: DESTINY

Test of Homogeneity of Variances

MMR

Levene Statistic	df1	df2	Sig.
4.983	2	1666	.007

Descriptives

MMR

	N	Mean	Std. Deviation	Std. Error	95% Confidence Interval for Mean		Minimum	Maximum
					Lower Bound	Upper Bound		
.00	660	.1727	.99219	.03862	.0969	.2485	-2.40	3.35
1.00	537	.1377	1.01737	.04390	.0514	.2239	-2.75	3.06
2.00	472	-.2964	.90383	.04160	-.3782	-.2147	-3.20	2.93
Total	1669	.0287	.99707	.02441	-.0191	.0766	-3.20	3.35

Contrast Tests

		Contrast	Value of Contrast	Std. Error	t	df	Sig. (2-tailed)
MMR	Assume equal variances	1	.3991	.10279	3.882	1666	.000
		2	-.0350	.05674	-.618	1666	.537
	Does not assume equal variances	1	.3991	.10456	3.817	990.696	.000
		2	-.0350	.05847	-.599	1134.143	.549

S2 Table 1-3: Descriptive statistics and ANOVA outputs for Destiny dataset

GAME: Battlefield 3

Test of Homogeneity of Variances

MMR

Levene Statistic	df1	df2	Sig.
1.816	2	8740	.163

Descriptives

MMR

	N	Mean	Std. Deviation	Std. Error	95% Confidence Interval for Mean Lower Bound	95% Confidence Interval for Mean Upper Bound	Minimum	Maximum
.00	3978	.1381	.98694	.01565	.1074	.1688	-3.31	3.38
1.00	2430	.1566	.94183	.01911	.1192	.1941	-3.31	3.08
2.00	2335	-.2206	.96661	.02000	-.2598	-.1814	-3.34	3.20
Total	8743	.0474	.98249	.01051	.0268	.0680	-3.34	3.38

ANOVA

MMR

	Sum of Squares	df	Mean Square	F	Sig.
Between Groups	229.421	2	114.711	122.128	.000
Within Groups	8209.184	8740	.939		
Total	8438.605	8742			

Contrast Tests

		Contrast	Value of Contrast	Std. Error	t	df	Sig. (2-tailed)
MMR	Assume equal variances	1	.3958	.04674	8.469	8740	.000
		2	.0186	.02495	.744	8740	.457
	Does not assume equal variances	1	.3958	.04588	8.627	4609.502	.000
		2	.0186	.02470	.752	5318.884	.452

S2 Table 4-6: Descriptive statistics and ANOVA outputs for Battlefield 3 dataset

GAME: League of Legends

Test of Homogeneity of Variances

MMR

Levene Statistic	df1	df2	Sig.
15.442	2	17858	.000

Descriptives

MMR

	N	Mean	Std. Deviation	Std. Error	95% Confidence Interval for Mean		Minimum	Maximum
					Lower Bound	Upper Bound		
.00	11125	-.0392	.97255	.00922	-.0573	-.0211	-2.66	3.52
1.00	5146	.1331	1.01132	.01410	.1055	.1607	-2.66	3.47
2.00	1590	-.0762	1.05834	.02654	-.1282	-.0241	-2.66	3.51
Total	17861	.0071	.99495	.00744	-.0075	.0217	-2.66	3.52

Contrast Tests

		Contrast	Value of Contrast	Std. Error	t	df	Sig. (2-tailed)
MMR	Assume equal variances	1	.3816	.03836	9.947	17858	.000
		2	.1723	.01672	10.306	17858	.000
	Does not assume equal variances	1	.3816	.03981	9.586	5760.801	.000
		2	.1723	.01685	10.228	9669.915	.000

S2 Table 7-9: Descriptive statistics and ANOVA outputs for LoL dataset

GAME: DOTA 2

Test of Homogeneity of Variances

MMR

Levene Statistic	df1	df2	Sig.
5.156	2	283	.006

Descriptives

MMR

	N	Mean	Std. Deviation	Std. Error	95% Confidence Interval for Mean		Minimum	Maximum
					Lower Bound	Upper Bound		
.00	107	-.1878	1.08060	.10447	-.3949	.0193	-2.92	1.93
1.00	143	.1865	.86779	.07257	.0430	.3300	-3.26	3.15
2.00	36	-.2006	1.12977	.18829	-.5829	.1816	-2.81	2.00
Total	286	-.0023	1.00101	.05919	-.1188	.1142	-3.26	3.15

Contrast Tests

		Contrast	Value of Contrast	Std. Error	t	df	Sig. (2-tailed)
MMR	Assume equal variances	1	.7614	.25168	3.025	283	.003
		2	.3743	.12609	2.968	283	.003
	Does not assume equal variances	1	.7614	.25968	2.932	113.215	.004
		2	.3743	.12720	2.943	198.484	.004

S2 Table 10-12: Descriptive statistics and ANOVA outputs for DOTA2 dataset

Means Plots

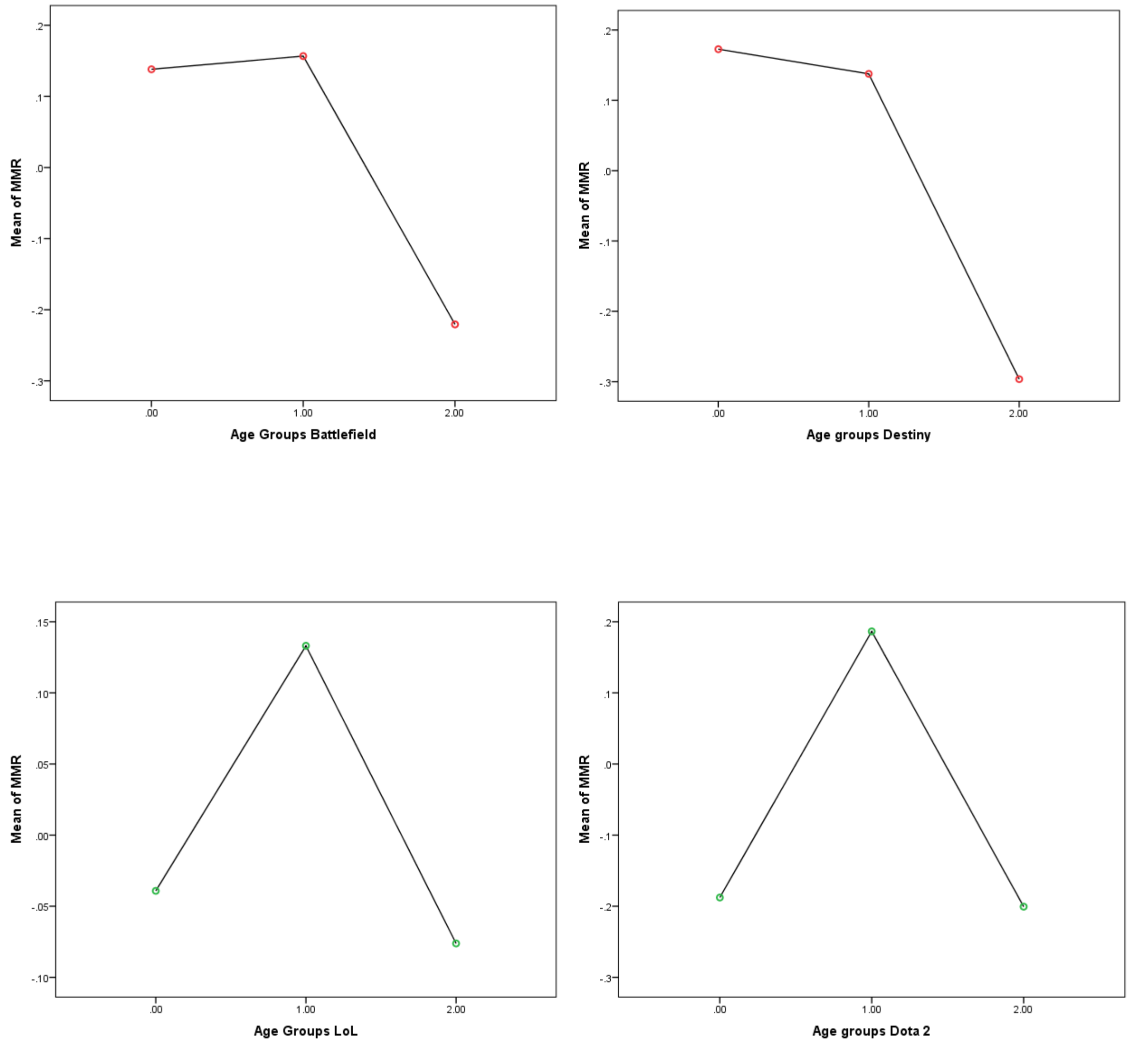

S2 Fig 1-4: Mean plots of MMR in each age group for four different games.
