# Supplementary material for: Exploring the relationship between video game expertise and fluid intelligence
Source: PLoS One. 2017 Nov 15;12(11):e0186621. doi: 10.1371/journal.pone.0186621 (PMC5687598; doi:10.1371/journal.pone.0186621)
Supplement: S3 File — (PDF) [file pone.0186621.s003.pdf]

# Supplementary Material – 3

Confirmatory factor analysis

# Confirmatory Factor Analysis

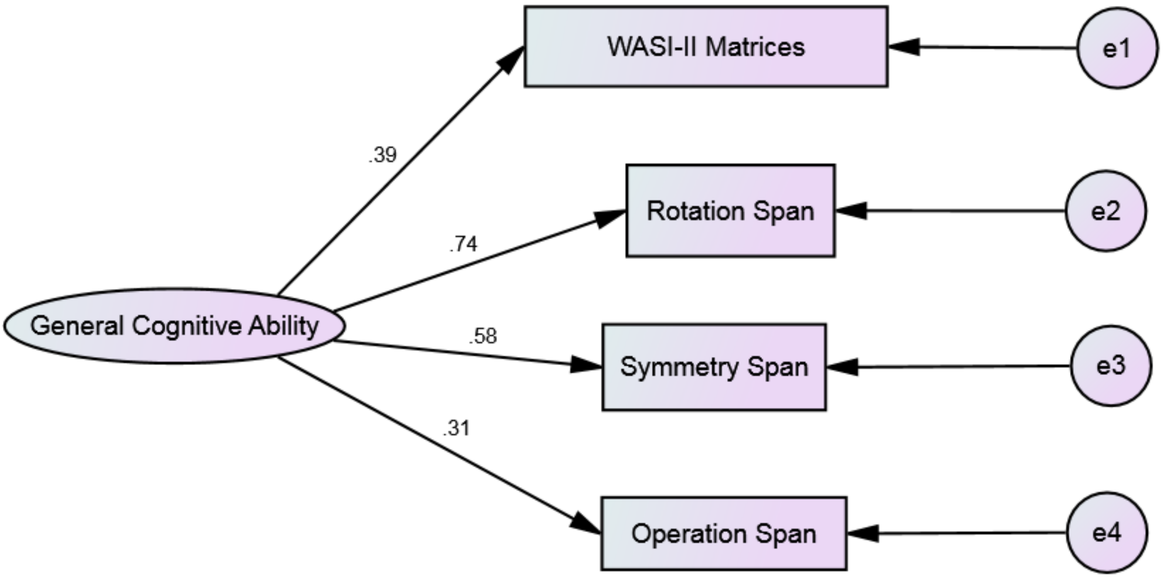


The following tests of significance and goodness-of-fit measures were obtained: $\chi^2(34) = 101.9$; $p<.001$

**Notes for Model (Default model)**

**Computation of degrees of freedom (Default model)**

| | |
|---|---|
| Number of distinct sample moments: | 10 |
| Number of distinct parameters to be estimated: | 8 |
| Degrees of freedom (10 - 8): | 2 |

**Result (Default model)**

Minimum was achieved
Chi-square = .009
Degrees of freedom = 2
Probability level = .995

The chi-square value associated with the model is **31.765** and, with **8** degrees of freedom, is statistically significant (*p*<.001).

**Model Fit Summary**

**CMIN**

| Model | NPAR | CMIN | DF | P | CMIN/DF |
|---|---|---|---|---|---|
| Default model | 8 | .009 | 2 | .995 | .005 |
| Saturated model | 10 | .000 | 0 | | |
| Independence model | 4 | 20.168 | 6 | .003 | 3.361 |

**RMR, GFI**

| Model | RMR | GFI | AGFI | PGFI |
|---|---|---|---|---|
| Default model | .175 | 1.000 | 1.000 | .200 |
| Saturated model | .000 | 1.000 | | |
| Independence model | 14.624 | .827 | .712 | .496 |

**Baseline Comparisons**

| Model | NFI Delta1 | RFI rho1 | IFI Delta2 | TLI rho2 | CFI |
|---|---|---|---|---|---|
| Default model | 1.000 | .999 | 1.110 | 1.422 | 1.000 |
| Saturated model | 1.000 | | 1.000 | | 1.000 |
| Independence model | .000 | .000 | .000 | .000 | .000 |

**Parsimony-Adjusted Measures**

| Model | PRATIO | PNFI | PCFI |
|---|---|---|---|
| Default model | .333 | .333 | .333 |
| Saturated model | .000 | .000 | .000 |
| Independence model | 1.000 | .000 | .000 |

**NCP**

| Model | NCP | LO 90 | HI 90 |
|---|---|---|---|
| Default model | .000 | .000 | .000 |
| Saturated model | .000 | .000 | .000 |
| Independence model | 14.168 | 4.142 | 31.768 |

**FMIN**

| Model | FMIN | F0 | LO 90 | HI 90 |
|---|---|---|---|---|
| Default model | .000 | .000 | .000 | .000 |
| Saturated model | .000 | .000 | .000 | .000 |
| Independence model | .367 | .258 | .075 | .578 |

**RMSEA**

| Model | RMSEA | LO 90 | HI 90 | PCLOSE |
|---|---|---|---|---|
| Default model | .000 | .000 | .000 | .996 |

| Model | RMSEA | LO 90 | HI 90 | PCLOSE |
|---|---|---|---|---|
| Independence model | .207 | .112 | .310 | .007 |

**AIC**

| Model | AIC | BCC | BIC | CAIC |
|---|---|---|---|---|
| Default model | 16.009 | 17.609 | 32.212 | 40.212 |
| Saturated model | 20.000 | 22.000 | 40.254 | 50.254 |
| Independence model | 28.168 | 28.968 | 36.269 | 40.269 |

**ECVI**

| Model | ECVI | LO 90 | HI 90 | MECVI |
|---|---|---|---|---|
| Default model | .291 | .327 | .327 | .320 |
| Saturated model | .364 | .364 | .364 | .400 |
| Independence model | .512 | .330 | .832 | .527 |

**HOELTER**

| Model | HOELTER .05 | HOELTER .01 |
|---|---|---|
| Default model | 35899 | 55185 |
| Independence model | 35 | 46 |

| | |
|---|---|
| Minimization: | .011 |
| Miscellaneous: | .201 |
| Bootstrap: | .000 |
| Total: | .212 |

**S3 Table 1-10: Raw output from the CFA analysis showing model fit.**
