# Supplementary material for: Exploring the relationship between video game expertise and fluid intelligence
Source: PLoS One. 2017 Nov 15;12(11):e0186621. doi: 10.1371/journal.pone.0186621 (PMC5687598; doi:10.1371/journal.pone.0186621)
Supplement: S4 File — (PDF) [file pone.0186621.s004.pdf]

Supplementary Material – 4

Correlations

Storage Correlations

Here we provide the correlation values for subsets of the psychometric data that were incorporated into Study 1. The variable names are as follows:

ROTATION_ERRORS: Number of errors in the Rotation Task (Participant did not correctly rotate a letter presented to them).

MATH_CORRECT: Number of simple arithmetic operations that the participant got correct.

SYMMETRY_CORRECT: Number of Correct Answers in the first part of the Symmetry Span task (Participant had to identify whether an image was symmetric or not).

WASI_RAW_SCORE: Raw score in the Matrix Subtest.

LITERAL_RANKINGS: League of Legends Rank based on the players tier and division (Silver 5 is lower than Silver 4 etc, Silver 1 is lower than Gold 5 etc).

ROT_SPAN_ABSOLUTE: Participants were awarded points if they remembered a full sequence of arrows correctly.

SYM_SPAN_ABSOLUTE: Participants were awarded points if they remembered a full sequence of red squares appearing with the confines of matrix correctly.

OS_SPAN_ABSOLUTE: Participants were awarded points if they remembered a full sequence of letters correctly.

Correlations

			ROTATION_ERRORS	MATH_CORRECT	SYMMETRY_CORRECT	WASI_RAW_SCORE	Literal_Ranking	ROT_SPAN_ABSOLUTE	SYM_SPAN_ABSOLUTE	OS_PAN_ABSOLUTE
Spearman's rho	ROTATION_ERRORS	Correlation Coefficient	1.000	-.371**	-.225*	-0.201	0.104	-.463**	-0.215	-0.140
		Sig. (1-tailed)		0.003	0.049	0.071	0.225	0.000	0.061	0.154
		N	55	55	55	55	55	51	53	55
	MATH_CORRECT	Correlation Coefficient	-.371**	1.000	.255*	0.174	0.091	.254*	0.106	0.094
		Sig. (1-tailed)	0.003		0.030	0.102	0.253	0.036	0.225	0.247
		N	55	55	55	55	55	51	53	55
	SYMMETRY_CORRECT	Correlation Coefficient	-.225*	.255*	1.000	0.000	0.115	.303*	0.124	.253*
		Sig. (1-tailed)	0.049	0.030		0.499	0.201	0.015	0.188	0.031
		N	55	55	55	55	55	51	53	55
	WASI_RAW_SCORE	Correlation Coefficient	-0.201	0.174	0.000	1.000	.440**	.303*	.247*	0.071
		Sig. (1-tailed)	0.071	0.102	0.499		0.000	0.014	0.036	0.302
		N	55	55	55	56	56	52	54	56
	Literal_Ranking	Correlation Coefficient	0.104	0.091	0.115	.440**	1.000	.260*	0.117	0.025
		Sig. (1-tailed)	0.225	0.253	0.201	0.000		0.031	0.199	0.427
		N	55	55	55	56	56	52	54	56
	ROT_SPAN_ABSOLUTE	Correlation Coefficient	-.463**	.254*	.303*	.303*	.260*	1.000	.440**	0.198
		Sig. (1-tailed)	0.000	0.036	0.015	0.014	0.031		0.001	0.080
		N	51	51	51	52	52	52	50	52
	SYM_SPAN_ABSOLUTE	Correlation Coefficient	-0.215	0.106	0.124	.247*	0.117	.440**	1.000	0.197
		Sig. (1-tailed)	0.061	0.225	0.188	0.036	0.199	0.001		0.077
		N	53	53	53	54	54	50	54	54
	OS_PAN_ABSOLUTE	Correlation Coefficient	-0.140	0.094	.253*	0.071	0.025	0.198	0.197	1.000
		Sig. (1-tailed)	0.154	0.247	0.031	0.302	0.427	0.080	0.077	
		N	55	55	55	56	56	52	54	56

**. Correlation is significant at the 0.01 level (1-tailed).

*. Correlation is significant at the 0.05 level (1-tailed).

S4 Table 1: Raw cross correlations for data in Study 1.
