# Supplementary material for: Exploring the relationship between video game expertise and fluid intelligence
Source: PLoS One. 2017 Nov 15;12(11):e0186621. doi: 10.1371/journal.pone.0186621 (PMC5687598; doi:10.1371/journal.pone.0186621)
Supplement: S5 File — (PDF) [file pone.0186621.s005.pdf]

Supplementary Material – 5

Advert and recruitment

Participants (N=56, 51 males, mean age 20.5 years) were recruited via adverts from multiple sites within the UK in and around the Universities of Leeds, Essex and York. All subjects were experienced LoL players who had played a large number (>100) of both 'ranked' and 'unranked' matches.

Subjects had normal or corrected to normal vision and reported no clinical psychiatric conditions. Consent forms confirmed that participants were willing to share their LoL data and that we were able to verify their ranks and numbers of games played.

A copy of the advert used to recruit subjects is provided below:

Exploring the relationship between video game expertise and fluid Intelligence

A.V. Kokkinakis, P. Cowling, A., Drachen, A.R. Wade

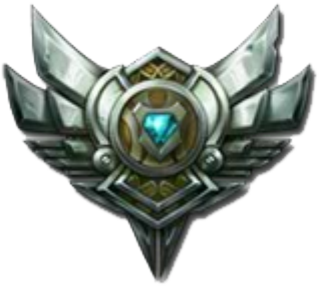

League of Legends Experiment

I am psychologist looking for participants who play League of Legends.

Participant Requirements:

- You must be over 18.
- You will be paid **£8** for 1 hour and 35 minutes.
- The experiment will be a series of working memory and problem solving tasks in addition to a personality questionnaire.
- When you come to the Psychology building to do the experiment your eyesight needs to be ok (wear glasses/contact lenses).
- You must have played a lot of ranked games **(over 100 at least)**.
- Your account must be in the following servers: NA, EU W, EU N&E, OCE or LAN.
- There is no rank requirement (any division will do).
