# Supplementary material for: Exploring the relationship between video game expertise and fluid intelligence
Source: PLoS One. 2017 Nov 15;12(11):e0186621. doi: 10.1371/journal.pone.0186621 (PMC5687598; doi:10.1371/journal.pone.0186621)
Supplement: S6 File — (PDF) [file pone.0186621.s006.pdf]

Supplementary Material – 6

Tasks

Visual depictions of the tasks are presented here for visualisation processes. See (Foster et al.,2014) (DOI 10.3758/s13421-014-0461-7) for the a more exact representation of the tasks.

OSPAN

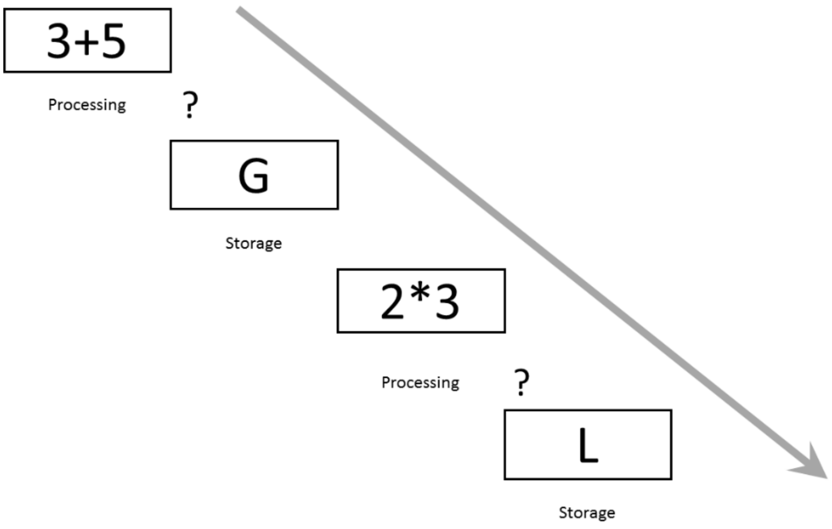

S6 Fig 1 – Visual depiction of the ospan task

SYMSPAN

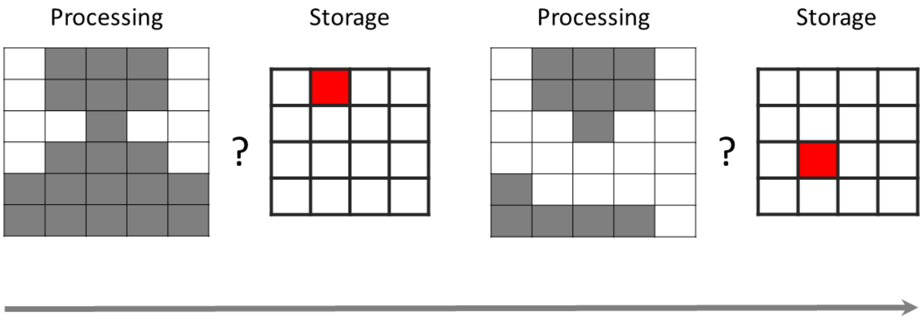

S6 Fig 2 – Visual depiction of the symspan task

ROTSPAN

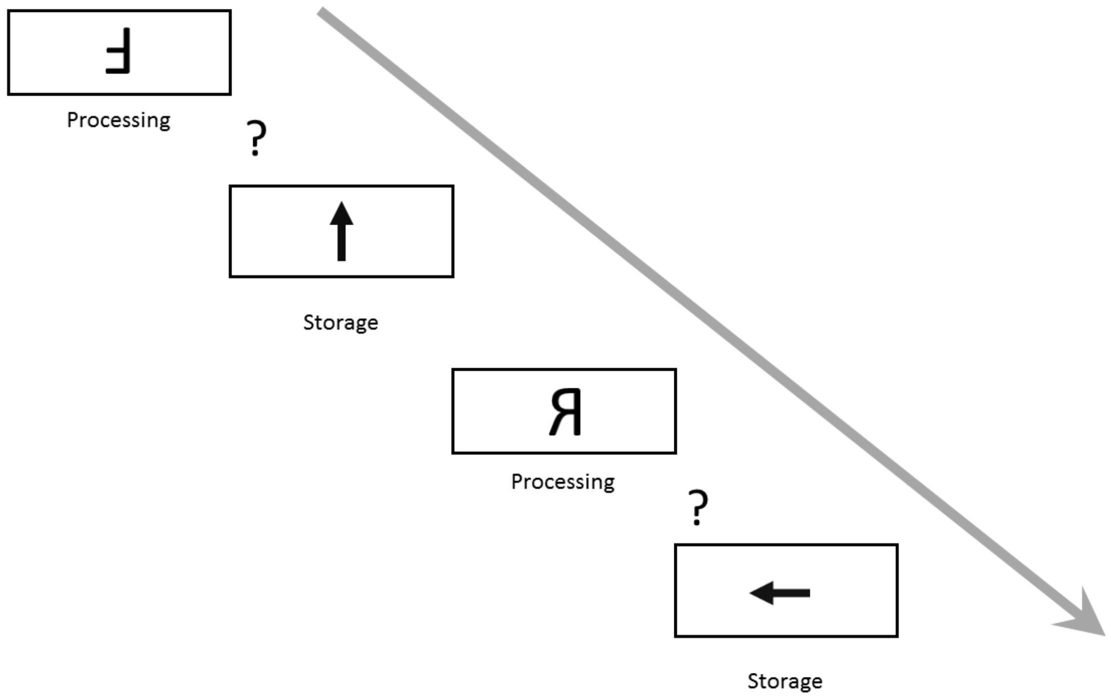

S6 Fig 3 – Visual depiction of the rotspan task
